# Synonymous mutations in the phosphoglycerate kinase 1 gene induce an altered response to protein misfolding in *Schizosaccharomyces pombe*

**DOI:** 10.3389/fmicb.2022.1074741

**Published:** 2023-01-11

**Authors:** Sandra Moreira-Ramos, Loreto Arias, Rodrigo Flores, Assaf Katz, Gloria Levicán, Omar Orellana

**Affiliations:** ^1^Programa de Biología Celular y Molecular, Instituto de Ciencias Biomédicas, Facultad de Medicina, Universidad de Chile, Santiago, Chile; ^2^Departamento de Biología, Facultad de Química y Biología, Universidad de Santiago de Chile (USACH), Santiago, Chile

**Keywords:** translation, codon usage bias, synonymous mutation, protein aggregation, proteostasis, stress tolerance

## Abstract

**Background:**

Proteostasis refers to the processes that regulate the biogenesis, folding, trafficking, and degradation of proteins. Any alteration in these processes can lead to cell malfunction. Protein synthesis, a key proteostatic process, is highly-regulated at multiple levels to ensure adequate adaptation to environmental and physiological challenges such as different stressors, proteotoxic conditions and aging, among other factors. Because alterations in protein translation can lead to protein misfolding, examining how protein translation is regulated may also help to elucidate in part how proteostasis is controlled. Codon usage bias has been implicated in the fine-tuning of translation rate, as more-frequent codons might be read faster than their less-frequent counterparts. Thus, alterations in codon usage due to synonymous mutations may alter translation kinetics and thereby affect the folding of the nascent polypeptide, without altering its primary structure. To date, it has been difficult to predict the effect of synonymous mutations on protein folding and cellular fitness due to a scarcity of relevant data. Thus, the purpose of this work was to assess the effect of synonymous mutations in discrete regions of the gene that encodes the highly-expressed enzyme 3-phosphoglycerate kinase 1 (*pgk1*) in the fission yeast *Schizosaccharomyces pombe*.

**Results:**

By means of systematic replacement of synonymous codons along *pgk1*, we found slightly-altered protein folding and activity in a region-specific manner. However, alterations in protein aggregation, heat stress as well as changes in proteasome activity occurred independently of the mutated region. Concomitantly, reduced mRNA levels of the chaperones Hsp9 and Hsp16 were observed.

**Conclusion:**

Taken together, these data suggest that codon usage bias of the gene encoding this highly-expressed protein is an important regulator of protein function and proteostasis.

## Introduction

Synonymous codons encoding the same amino acid, were long thought to be equivalent and interchangeable. Therefore, synonymous mutations were often called “silent.” However, a growing body of evidence has shown that synonymous codon choice in nature is not random and may affect multiple aspects of protein biogenesis in diverse organisms (Chen et al., 2004; Chaney and Clark, 2015; Chaney et al., 2017). Usage frequencies of synonymous codons vary greatly between species and within the same genome, a phenomenon called codon usage bias (CUB). In fast-growing unicellular organisms, the most-frequent codons (called optimal codons) are usually decoded by highly-expressed tRNAs, and the less-frequent codons (called rare or non-optimal codons) are decoded by less-expressed tRNAs (Ikemura, 1985; Kanaya et al., 1999; Quax et al., 2015).

Each organism has a defined CUB, which has been modeled by the co-evolution of the tRNA genes and the mutational rate of the coding genes. In fast-growing organisms, tRNA gene copy number is generally correlated with the abundance of the corresponding tRNA (Ikemura, 1985). In these organisms, CUB can lead to translation optimization or de-optimization of a group of related mRNAs, coordinating their expression (Zhou et al., 2016). Moreover, CUB has been invoked as a major factor in determining both mRNA and protein levels (Zhou et al., 2016). However, the extent to which translation efficiency of an mRNA relates codon bias to protein levels remains unclear.

In recent years, the issue of CUB has become even more relevant as it has been linked to many processes that affect gene expression, such as mRNA stability, translation accuracy as well as protein levels, folding and localization (reviewed in Drummond and Wilke, 2008; Zhang and Ignatova, 2009; Zhang et al., 2010; Subramaniam et al., 2013; Pechmann et al., 2014; Bali and Bebok, 2015; Buhr et al., 2016; Thommen et al., 2017; Bhattacharyya et al., 2018; Hanson and Coller, 2018). However, it is controversially discussed because while it has been established that codon usage is an important factor in estimating protein concentration in yeast (Hiraoka et al., 2009) and trypanosomatids (Jeacock et al., 2018), in humans it has been claimed that translation elongation speed is independent of CUB (Ingolia et al., 2011).

Of the processes mentioned above, the effect of codon choice on codon reading speed is one the least understood. Since protein folding is a co-translational process for many proteins, codon changes that affect codon reading speed can also alter the folding of the encoded protein, leading to abnormal protein functioning (reviewed in Hunt et al., 2014; Chaney and Clark, 2015; Brule and Grayhack, 2017; Rauscher and Ignatova, 2018; Walsh et al., 2020). For example, the synonymous substitution of five low-usage for high-usage codons in a linker region of the fatty acid-binding protein 1 gene in *Echinococcus granulosus* was shown to affect protein solubility (Cortazzo et al., 2002). Also, it was demonstrated that replacement of synonymous codons in the gene encoding *Bos. taurus* gamma-B crystallin affects the translation rate, generating different final folding forms of the protein when expressed in *Escherichia coli* (Buhr et al., 2016). Moreover, the synonymous polymorphisms found in a common haplotype [at 1236C > T (G412G), 2677G > T (A893S), and 3435C > T (I1145I)] in the human multidrug resistance 1 (MDR1) gene have been associated with multidrug resistance shown by cancer cells. This phenotype is attributable to an impairment of folding and activity of the protein, without altering mRNA and protein levels (Kimchi-Sarfaty et al., 2007; Fung and Gottesman, 2009). Recently, Walsh et al. (2020) demonstrated in *E. coli* that synonymous codon substitutions in the chloramphenicol acetyltransferase gene alter the translation elongation speed, affecting protein folding and leading to an active protein that is more susceptible to degradation. The effect of codon usage in different regions of the protein (e.g., linker region, protein domains, etc.) is still unknown, although it has been proposed that regions between protein domains are encoded by non-optimal codons, while structured domains are encoded by optimal codons (Zhou et al., 2015).

Given the above, and since alterations in the folding of one protein might affect the response of the cell to this abnormality and/or induce modifications in the folding or aggregation of other proteins, it is important to understand the contribution of codon selection on the regulation of protein homeostasis. To add to our knowledge of these processes, we studied the effects of synonymous mutations in the gene encoding 3-phosphoglycerate kinase (*pgk1*) from the fission yeast *Schizosaccharomyces pombe* on the properties of the encoded protein (Pgk1), proteostasis, and cell fitness. *S. pombe* is a model unicellular eukaryotic organism that has been used in the study of mechanisms involved in transcription, translation, and replication (Hoffman et al., 2015). Pgk1 (EC 2.7.2.3) is a highly-expressed monomeric protein that catalyzes the reversible conversion of 1,3-bisphosphoglycerate (1,3-BPGA) to 3-phosphoglycerate (3-PGA) in glycolysis (Banks et al., 1979; Bowler, 2013), and is one of the only two enzymes that generate ATP at the substrate level in this pathway. We selected this gene as a model because (i) almost all codons are optimal, (ii) CUB of *pgk1* is conserved among several organisms, and (iii) the gene is highly-expressed. Previous work performed in *pgk1* from *S. cerevisiae* showed that the replacement of a large number of codons with their synonymous counterparts affected mRNA stability and Pgk1 expression (Hoekema et al., 1987); however a detailed analysis of the contribution of each synonymous mutation to the observed results and their effect on yeast physiology remain unexplored.

In this work, we introduced synonymous mutations in short segments along the entire gene encoding Pgk1 and studied the effect of each mutated segment on the encoded protein and its impact on the organism. Depending on the mutated region, we observed subtle effects at the molecular level; however, independent of their location, all mutations altered protein aggregation without affecting Pgk1 levels. Also, some mutated *pgk1* changed the response of the cell to heat and misfolding stress. The results obtained in the present study will help to elucidate the role of codon choice on protein aggregation and homeostasis, leading to a better understanding of the implications of synonymous mutations for cell physiology.

## Results

### The *pgk1* gene shows a strong codon usage bias for optimal codons

The protein sequence of *S. pombe* Pgk1 is 68.36 and 67.07% identical to those of *Saccharomyces cerevisiae* (PDB: 1QPG_A) and *Homo sapiens* (PDB: 4AXX_A) respectively, with 99% coverage. Alignments of primary sequences and comparisons of secondary structures revealed a high degree of conservation between these three proteins (Supplementary Figure 1).

We calculated the codon adaptation index (CAI) and tRNA adaptation index (tAI) for the *pgk1* gene from *S. pombe* (Sharp and Li, 1987; Dos Reis et al., 2004). The score measured by CAI ranges from 0 to 1, such that when the CAI value is higher (closer to 1), the gene is more likely to be highly-expressed. The CAI values revealed that most *pgk1* codons are optimal, similar to those of genes that codify the most abundant proteins (e.g., ribosomal proteins) (Table 1; Mehdi, 2014; Quax et al., 2015). Only 37 of the 414 codons in the *pgk1* sequence are non-optimal (9%), of which 15 are found proximal to the 150 codons encoding the carboxyl terminus of the protein. We found no obvious preference of non-optimal codons within specific regions of the protein (e.g., linkers between domains or inside domains). Additionally, the tAI values indicate that codons present in *pgk1* are decoded by tRNAs with high gene copy number, a finding which is concordant with the CAI score. Thus, we conclude that *pgk1* codon usage is optimized, as in most highly-expressed proteins. Altogether, these results indicate that *pgk1* has a strong bias to use optimal codons, as found in other species, such as unicellular organisms with high proliferation rates (Supplementary Tables 3, 4). In this study, we did not include an analysis of higher eukaryotes genes, because neither global tAI nor CAI are reliable predictors of codon optimality in these multicellular organisms since the tRNA and mRNA expression levels are tissue-specific (Waldman et al., 2010).

**Table 1:** Codon adaptation index (CAI) and tRNA adaptation index (tAI) of *Schizosaccharomyces pombe* genes.

Protein encoded	CAI	tAI	Gene ID
Pgk1	0.861	0.3980	2539764
60S ribosomal protein L2	0.79	0.3539	2539780
RNA polymerase III transcription factor TFIIIA	0.383	0.1625	2542887
60S acidic ribosomal protein Rpp0	0.768	0.5930	2539764
60S ribosomal protein L9	0.699	0.5901	2539464

The Gene ID corresponds to the number of the gene assigned in the NCBI database.

**Figure 1:**
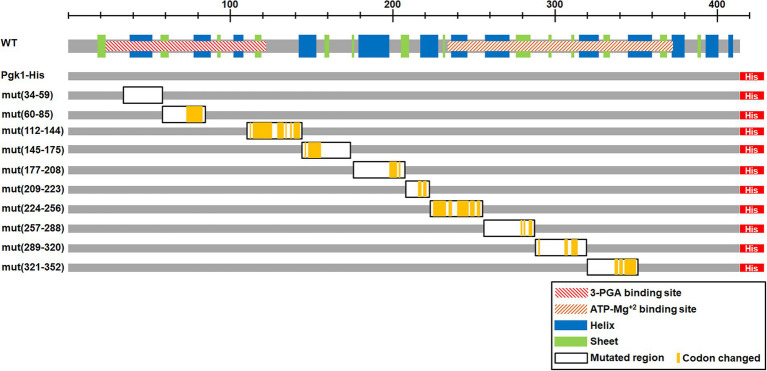
Scheme of the mutated regions. The upper scheme presents the Pgk1 protein (in gray). The hatched rectangles indicate the protein domains predicted using the Pfam protein family database [3-phosphoglycerate (3-PGA) binding site and ATP-Mg^2+^ binding site]. Blue and green rectangles indicate α helices and β strands, respectively. Lower schemes indicate the different constructions, with the 7xHis tag at the C-terminus (His, red rectangle). The left column indicates the name and position of the mutated codons (WT correspond to the control without mutations, the numbers indicate the mutated region). Black framed rectangles indicate the mutated regions and yellow lines show the actual mutated codons.

To test the potential role of the optimal codon bias in the expression and/or function of Pgk1, we randomly replaced wild-type optimal codons with synonymous non-optimal counterparts (based on the codon use of *pgk1* and CUB of *S. pombe*) in 10 discrete segments covering the entire *pgk1* gene (Figure 1).

### Synonymous mutations located in the linker region alter growth rate

To assess the effect of synonymous codon mutations on the expression and function of Pgk1, 10 independent segments of *pgk1* were mutated and replaced in the chromosome by homologous recombination (Figure 1). Thus, the mutated versions of *pgk1* remain governed by the wild-type (WT) promoter and regulatory sequences, thereby maintaining the same genetic context as WT *pgk1*. We discarded the use of an ectopic vector for overexpression of *pgk1* because higher levels of an artificially-produced protein may cause aggregation (Fink, 1998; Baneyx and Mujacic, 2004). The codons to be replaced were selected using the tabulations of codon usage provided in the study of Forsburg (Forsburg, 1994; Hiraoka et al., 2009). WT codons (optimal codons in general) were substituted by their non-optimal counterparts by random mutation at their third position in the selected regions, using the primers detailed in Supplementary Table 2. The positions of codon substitutions are indicated in Figure 1 (yellow bars). In the case of amino acids encoded by six synonymous codons (leucine, serine, and arginine), we also mutated the first position to randomly produce all codons with the lowest codon usage. As expected, tAI values for the described mutated sequences were lower than those of the control (Table 2). The DNA sequences of the mutated segments of *pgk1* are shown in Table 2. To easily identify the WT and mutated Pgk1 proteins, we introduced a region coding for a His-tag at the 3′ end of each *pgk1* gene (WT and mutants). The WT *pgk1* plus a His-tag (Pgk1-His WT) was used as a control and named WT. This strain showed the same characteristics as the parental strain in terms of morphology and growth (data not shown). Mutant strains were named mut (xxx-yyy) where xxx and yyy are the position of the first and the last mutated codons in each region, respectively.

**Table 2:** Wild-type and mutant sequences and tAI calculation of the corresponding mutated regions.

Mutant strain	Wild-type sequence	Mutant sequence	tAI of the wild-type segment	tAI of the mutant segment
mut(60–85)	TTGATGTCTCACTTGGGCC	TTGATGTCTCACTTGGGCC	0.5590	0.4904
	GTCCCAACGGTGCTCGTGT	GTCCCAACGGTGCTCG **C** GT		
	TGCCAAGTACTCCTTGAAG	TGCCAA **A** TACTCCTTGAA **A**		
	CCTGTTGCTGCTGAGCTCAGC	CC **C** GTTGCTGCTGAGCTCAGC		
mut(112–144)	GGTGGTGAGGTCATCCTTT	GGTGG **A** GAGGTGAT **A** CT **A** T	0.5801	0.4141
	TGGAGAACTTGCGTTTCCA	T **A** GA **A** AA **TC** T **A** CG **A** TT **T** CA **T**		
	CATCGAGGAGGAGGGTTC	AT **A** GA **A** GAGGAGGGTTC **T** G		
	CGCCAAGGTTGACGGTAAG	C **G** AA **A** GT **G** GACGG **C** AAGAA		
	AAGGTTAAGGCTGACGCTT	GGT **G** AAGGC **G** GA **T** GC **A** TCT		
	CTGCC	GCC		
mut(145–175)	GTCGAGGCTTTCCGTAAGT	GTCGAGGC **C** TTCCG **C** AA **A** T	0.4935	0.4261
	CTTTGACTTCTCTCGGTGAC	C **A** CTGAC **C** TC **A** CT **G** GG **G** GAC		
	ATCTTTGTCAACGATGCTTT	ATCTTTGTCAACGATGCTTTC		
	CGGTACCGCTCACCGTGCTC	GGTACCGCTCACCGTGCTCA		
	ACTCCTCTATGGTCTCTGCC	CTCCTCTATGGTCTCTGCC		
mut(321–352)	TTTGCCGAGGTTATCACCAC	TTTGCCGAGGTTATCACCAC	0.4413	0.4117
	CTCCAAGACCATTGTCTGGA	CTCCAAGACCATTGTCTGGA		
	ATGGTCCCGCTGGTGTCTTT	ATGGTCCCGCTGG **G** GT **G** TTT		
	GAGTTTGACAACTTTGCCAA	GA **A** TT **C** GACAA **T** TT **C** GC **T** AA		
	GGGTACCAAGTCTATG	**A** GG **G** AC **G** AA **A** TCTATG		

Underlined bold letters indicate the mutated nucleotides.

**Figure 2:**
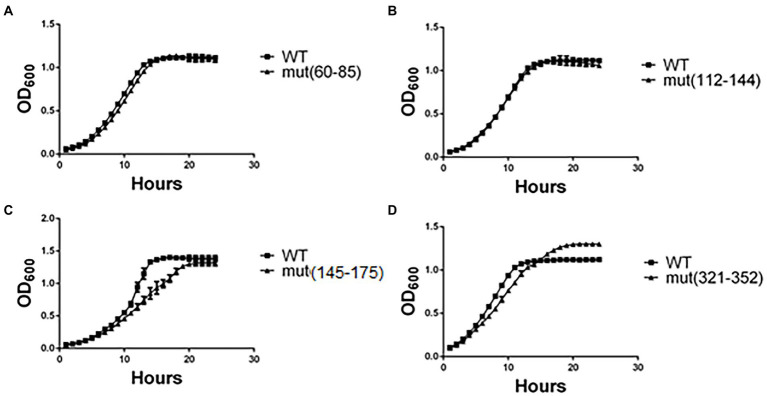
Yeast growth in minimal medium is altered in certain strains harboring synonymous mutations. Yeasts were grown in minimal medium (EMM2) under standard conditions for 24 h at 30°C, and OD_600_ recorded each hour. Readings of **(A)** mut(60–85), **(B)** mut(112–142), **(C)** mut(145–175), and **(D)** mut(321–352) were plotted and compared to the control (WT). These results are representative of three independent experiments.

As a first approach to assess the effect of synonymous mutations, we determined the cellular fitness (measured as specific growth rate, μ index, under standard conditions) of the mutant strains. Five to ten clones of each mutated region were randomly selected and grown in minimal medium (EMM2). Growth curves of representative clones are shown in Figure 2 (see version of this figure in logaritmic scale in Supplementary Figure 4), whilst the μ index and generation times are shown in Table 3. The generation time of most mutant *pgk1* strains (Table 3) was not affected, as seen in the cases of mut(60–85) and mut(112–144) (Figures 2A,B). However, mut(145–175) and mut(177–208) grew slower than the control (Figure 2C and Table 3). mut(321–352) possessed a μ index and generation time similar to the control but with an altered growth curve (Figure 2D and Table 3). No changes were observed in the mutants under microscopic observation or in terms of cell viability (data not shown). Altogether, the different mutations in discrete regions of *pgk1* showed no effect on cell growth, except in the cases of mutants mut(145–175), mut(177–208) and to some extent mut(321–352).

**Table 3:** Specific growth rate and generation time of WT and mutant strains.

Strains	μ (h^−1^)	Generation time (*h*)
Pgk-His WT	0.2359 ± 0.0250	2.91 ± 0.310
mut(34–59)	0.2642 ± 0.0012	2.62 ± 0.012
mut(60–85)	0.2306 ± 0.0144	3.02 ± 0.186
mut(112–144)	0.2366 ± 0.0109	2.93 ± 0.133
mut(145–175)	0.1659 ± 0.0036	4.18 ± 0.091
mut(177–208)	0.1955 ± 0.0002	3.54 ± 0.046
mut(209–233)	0.206 ± 0.0269	3.40 ± 0.478
mut(224–256)	0.2170 ± 0.0049	3.19 ± 0.073
mut(257–288)	0.2326 ± 0.0006	2.98 ± 0.008
mut(289–320)	0.2307 ± 0.0106	3.01 ± 0.137
mut(321–352)	0.2348 ± 0.0531	3.08 ± 0.70

Specific growth rate is expressed as μ index (h^−1^), and generation or doubling time in hours. Standard errors are shown.

Synonymous mutations harbored by mut(145–175) and mut(177–208) are located in the linker region between the 3-PGA-and ADP-Mg^2+^-binding domains and the hinge region, respectively. In the case of mut(321–352), the mutations are located within the ADP-Mg^2+^-binding domain (Figure 1). For further analyses, we selected two mutant strains that showed altered growth [mut(145–175) and mut(321–352)] and two strains with no change in cell growth [mut(60–85) and mut(112–144)].

### Synonymous mutations in *pgk1* do not alter protein levels but affect protein aggregation and enzyme activity

**Figure 3:**
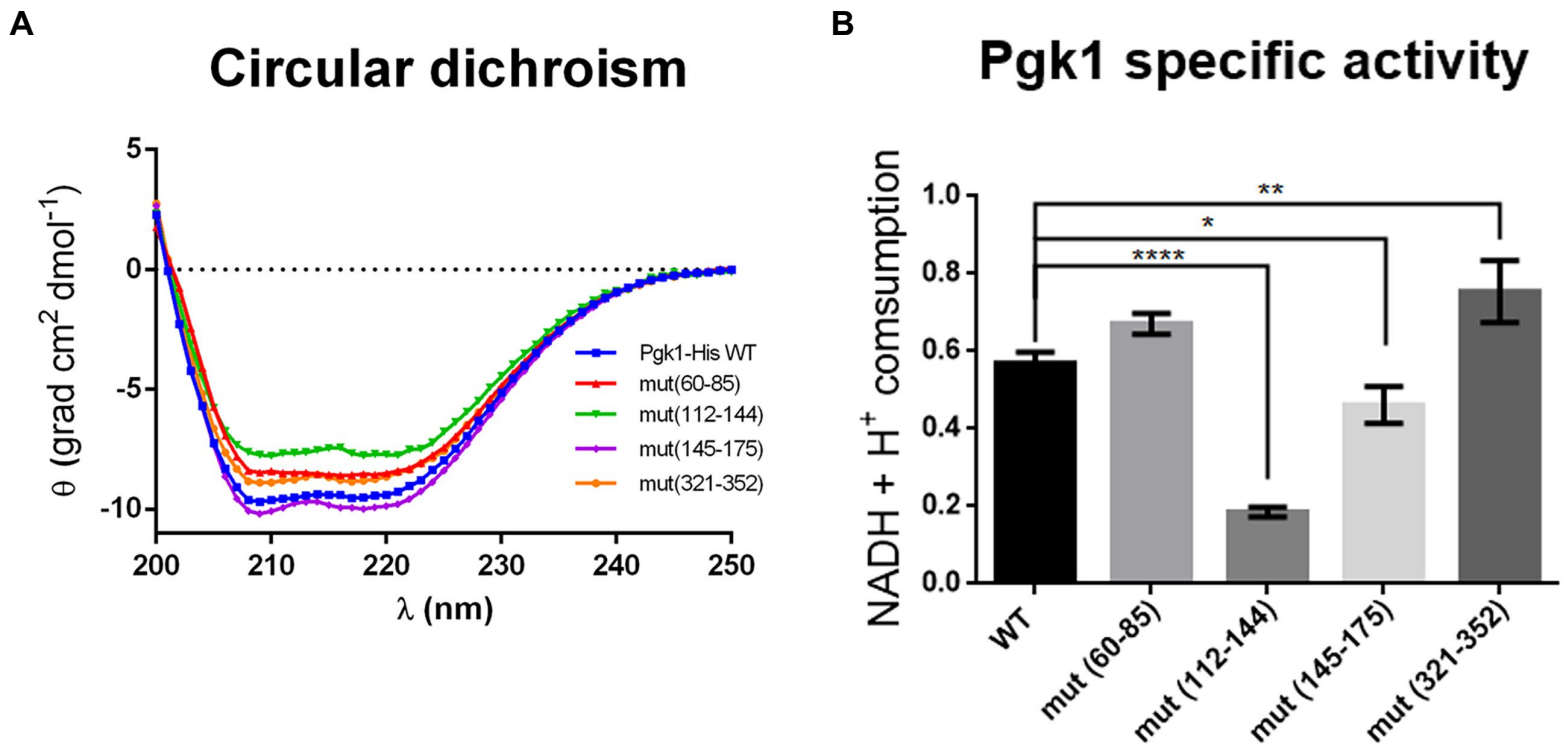
Pgk1 folding and activity are slightly altered by synonymous mutations in *pgk1*. **(A)** Circular dichroism of the purified Pgk1 from WT and mutant strains. **(B)** Pgk1 specific activity assayed with 5 nM purified Pgk1 (WT and mutants), in an assay coupled to GAPDH. The results are representative of three independent assays. Data was analyzed by one-way ANOVA (*p < 0.05; **p < 0.02; ****p < 0.0001).

To assess whether synonymous mutations alter the expression of Pgk1, we first measured the levels of *pgk1* mRNA. We observed an increase (with low statistical significance) in the mRNA levels of mut(145–175) and mut(321–352), but not the other two mutant strains (Supplementary Figure 2A). However, the levels of Pgk1 protein in all mutant strains were not altered compared to WT (Supplementary Figure 2B). The increase in the mRNA levels for mut(145–175) and mut(321–352) was thus not reflected in an expected increase in the protein levels, indicating that the translation efficiency of these mRNAs may be lowered as a result of the synonymous mutations. One possible explanation for this finding could be that an altered secondary structure of mutant mRNAs might increase their stability but concomitantly decrease their translation efficiency (Hunt et al., 2014). To test whether these synonymous mutations could modify the folding of the encoded proteins, we analyzed their secondary structure using the circular dichroism (CD) spectra of the soluble fraction of Pgk1 (WT and mutants) (Figure 3A). CD analysis of the purified proteins showed a predominant alpha-helix pattern in the control (WT) sample (Greenfield, 2006). Synonymous mutations in codons 60–85, 112–144, or 321–352, but not in codons 145–175, resulted in slightly altered CD spectra. Of the mutants, the spectra of mut(112–144) was the most affected, indicating a probable loss of α-helix content (Greenfield, 2006). In addition, we performed a protease sensitivity assay to estimate the folding status (Angov et al., 2011), obtaining variable results. Pgk1-His mut(60–85) was insensitive to trypsin, but mut(112–142), mut(145–175), and mut(321–352) were slightly sensitive (Supplementary Figure 3). Taken together, these data suggest that synonymous mutations cause subtle changes in the folding of Pgk1 in a manner that is dependent on the mutated region.

Given that the activity of a protein depends on its structure, we tested whether synonymous mutations produce alterations in the phosphorylation activity of 3-PGA by Pgk1. Activity of purified Pgk1-His from WT and mutant strains was analyzed in an enzymatic assay coupled to glyceraldehyde-3-phosphate dehydrogenase (GAPDH). Pgk1 from strains mut(60–85), mut(145–175), and mut(321–352) showed no change in enzymatic activity compared to WT. However, a marked 60–70% decrease in activity was observed for mut (112–144) (Figure 3B). This result correlates with the result that this strain has the most-affected CD spectrum (Figure 3A). Indeed, mut(112–144) harbors a large number of non-optimal substitutions (22 in total), 12 of which are found in tandem. The fact that these mutations are located in the linker between the two substrate-binding domains (Figure 1) led us to speculate that they might influence the translation rate of this segment, thus altering the folding of the protein.

Taken together, the data obtained thus far show that synonymous mutations cause slight structural variations in this soluble enzyme that may be reflected in changes in protein folding and activity. However, these alterations in the enzymatic activity of Pgk1 had no effect on the O_2_ consumption rate of the mutant strains (data not shown). This is probably because glycolysis is unaltered in the mutants since the reaction catalyzed by Pgk1 is not a limiting step in the pathway (Bolaños et al., 2008).

### Mutations in the *pgk1* gene increase protein aggregation

**Figure 4:**
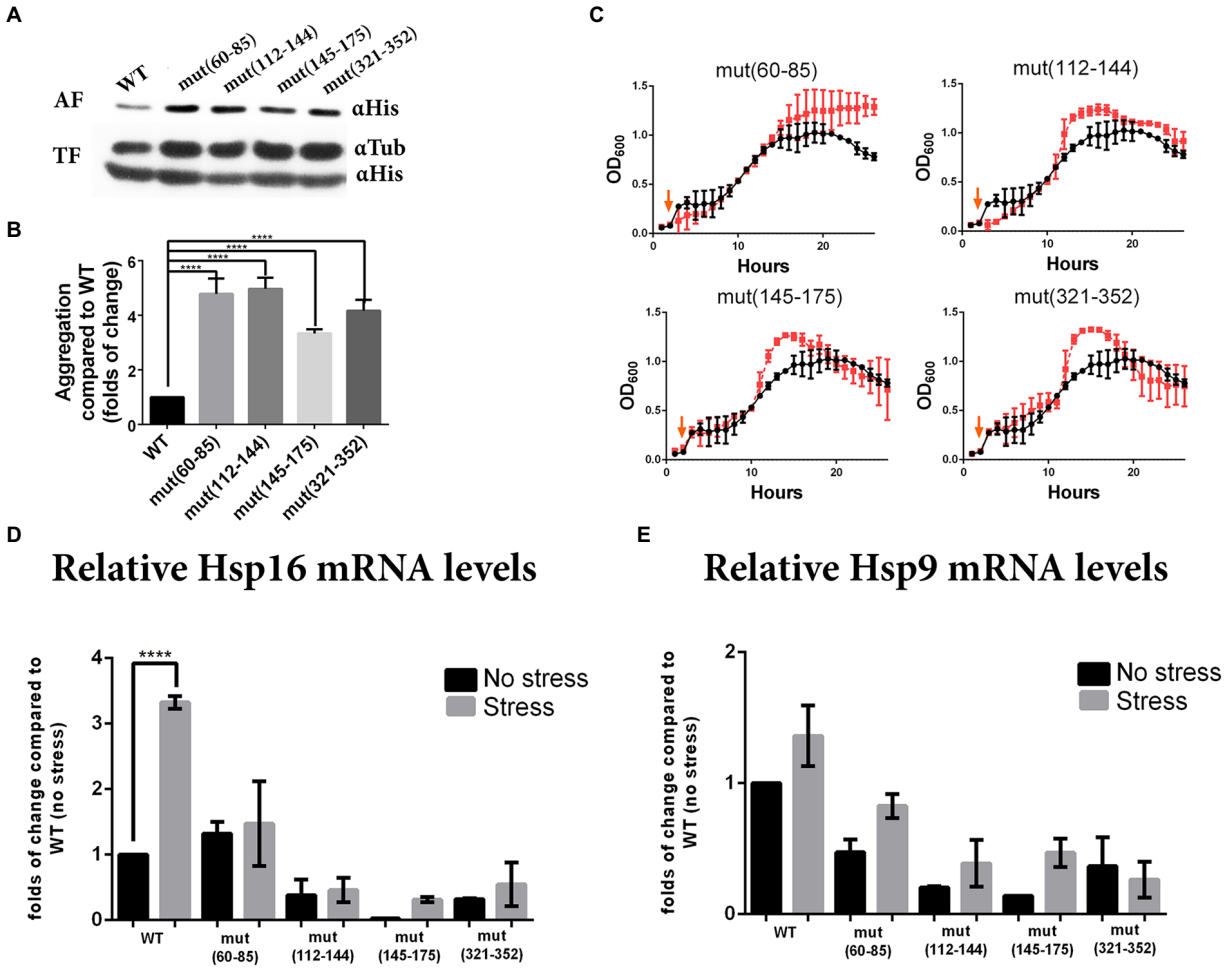
Synonymous mutations in *pgk1* alter protein aggregation, response to heat stress and chaperone expression. **(A)** Western blot of WT and mutants (αHis) from aggregated (AF) and total (TF) protein fractions isolated from yeasts cultured in EMM2 during the late logarithmic phase in standard conditions. **(B)** Quantification of the aggregated fraction (aggregated Pgk1 normalized to total Pgk1) and expressed as fold-change compared to WT. Data was analyzed by one-way ANOVA (****p < 0.0001). **(C)** Response to heat stress. Yeasts were grown in EMM2 at 42°C for 2 h, then shifted to 37°C (chronic heat stress, indicated with orange arrow) for 24 h. Black circles and red squares represent WT and mutant strains, respectively. **(D,E)** Quantification by real-time PCR of the levels of chaperones Hsp16 **(D)** and Hsp19 **(E)** mRNAs from WT and mutants, grown under standard conditions (30°C for 24 h, no stress, black bars) or heat stress condition (2 h at 42°C and 24 h at 37°C, stress, gray bars). The results are representative of three independent experiments, and data was analyzed by two-way ANOVA (*p* < 0.005).

Because folding is a co-translational process for most proteins (Fedorov and Baldwin, 1995; Thommen et al., 2017), we examined whether synonymous mutations in the selected regions could induce Pgk1 aggregation. We determined that 1–2% of total WT Pgk1 is normally found as an aggregate (Figure 4A, quantified in Figure 4B). We observed that all synonymous mutations increased protein aggregation, reaching up to 10% in the case of mut (112–144) (Figure 4A, quantified in Figure 4B). Therefore, a reduced translation rate caused by the replacement of optimal codons by non-optimal counterparts, or a different secondary structure of the mRNA at the mutated sites might induce an altered co-translational protein folding regime in which perhaps the concomitant increase in exposure of buried hydrophobic amino acids might induce an increase in protein aggregation, irrespective of the mutated region (Nackley et al., 2006; Chen et al., 2014).

### Synonymous mutations modify the response of cells to protein aggregation

**Figure 5:**
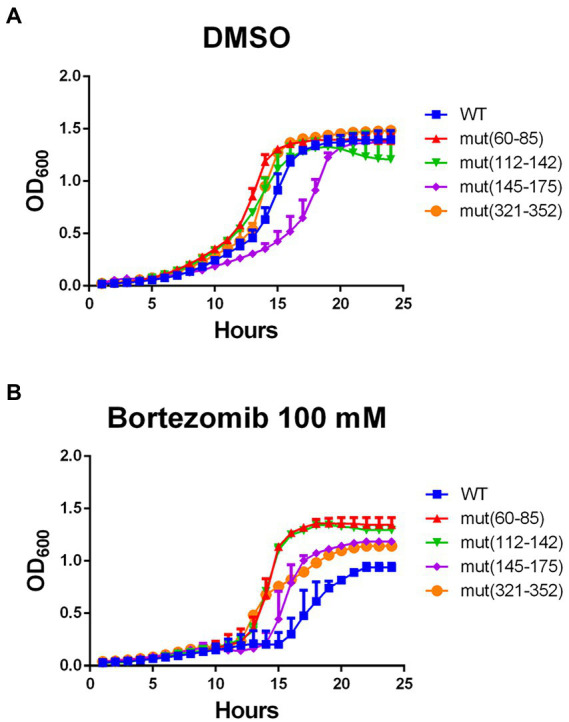
Response to the proteasome inhibitor bortezomib is improved by synonymous mutations in *pgk1*. Yeasts were incubated with vehicle (DMSO) **(A)** or 100 μM bortezomib **(B)** in EMM2 for 24 h in standard conditions. Blue squares, red triangles, green inverted triangles, purple diamonds, and orange circles represent WT, mut(60–85), mut(112–144), mut(145–175), and mut(321–352) respectively. Growth was monitored by recording OD_600_ at each hour.

Since WT *pgk1* was replaced in the genome by the mutant variants, we envisioned that the cells of the latter should be chronically exposed to an increase in aggregated Pgk1. Thus, we analyzed whether this phenomenon affects protein homeostasis. We tested the cellular response to bortezomib, a proteasome inhibitor that increases protein aggregation (Canfield et al., 2006; Mathiassen et al., 2015). All selected strains harboring synonymous mutations (except mut145–175) grew at a similar rate as the control (vehicle of bortezomib, Figure 5A). Although bortezomib at 100 μM affected cell growth in the WT strain (compare Figure 5A with Figure 5B). See version of these figures in logaritmic scale in Supplementary Figure 6, all mutant strains grew better than this control (Figure 5B). Proteasome activity was measured in control and mutant cells (Table 4) revealing a slight increase (average 10%) in all mutants [except mut(112–144)].

**Table 4:** Proteasome activity.

Strains	Proteasome activity (units)
WT	298.40 ± 1.58
mut(60–85)	342.91 ± 3.37
mut(112–144)	295.04 ± 1.06
mut(145–175)	314.70 ± 1.98
mut(321–352)	330.54 ± 3.05

Protein extracts (1 mg) from wild type (WT) and mutant strains were used in the assay in the presence of AMC-tagged peptide as substrate which released free highly-fluorescent AMC due to proteasome activity. Excitation/emission at 350/440 nm were measured at 37°C for 25 min. One unit of proteasome activity was calculated as the amount of proteasome which generates 1 nmol of product per minute at 37°C. The data shown are the means of three replicates ± SE.

Bortezomib is an inhibitor of the chymotrypsin-like subunit of the proteasome. We speculate that in the mutant cells, the chronic aggregation of Pgk1 might permanently increase the activity of the proteasome, giving rise to resistance of the cell to the drug, although other proteosomal functions might also be influenced by the mutant *pgk1* (Canfield et al., 2006; Takeda et al., 2011). Taken together, we speculate that the chronic exposure of the cells to aggregated Pgk1 in the synonymous mutant strains might improve their adaptation to environmental stress.

### Synonymous mutations alter the response of cells to stress

Since all the strains analyzed showed altered protein aggregation, and the response to proteasome inhibitor was opposite to that expected, we hypothesized that synonymous mutations might alter the cellular response to other stresses. To test whether synonymous mutations alter cell growth under heat stress, we exposed the cells to 42°C for 2 h followed by mild heat stress over a longer period to allow growth (37°C for 24 h) (Chung et al., 1998; Petersen and Russell, 2016). Under these conditions, most mutant strains showed a slight increase in growth rate during the logarithmic phase, yet all reached a higher OD_600_ at the stationary phase (Figure 4C). See a version of this figure in logaritmic scale in Supplementary Figure 5 than the control (WT) strain.

In the *S. pombe* genome, there are at least 17 members of HSP protein families that respond to different stress elicitors, where both chaperones Hsp9 and Hsp16 are highly expressed under heat treatment (Chen et al., 2003). For this reason, we measured mRNA levels of Hsp9 and Hsp16. As expected we observed that under heat stress, levels of Hsp16 mRNA increased up to 3-fold in WT control (Figure 4D). However Hsp9 mRNA showed no statistically significant increase (Figure 4E). Conversely, mRNA levels of both chaperones were lower in the mutant [except mut(60–85)] than in WT strains under both control and stress conditions (Figures 4D,E).

Taken together, as under heat shock the mutant strains grow better than WT, yet the expression of HSP mRNAs is the opposite as expected, we speculate that the exposure of the cells to aggregated Pgk1 might alter the heat shock stress response, reducing the levels of chaperone mRNA. Therefore, since mutant strains respond better than the WT to heat shock, grew better under proteasome inhibition and during the stationary phase (which is an inherently-stressful condition in yeast (Gray et al., 2004; Narayanaswamy et al., 2009; reviewed in Cotto and Morimoto, 1999), we speculate that the chronic exposure to aggregated Pgk1 alerts the mutant cells, enhancing their pre-adaptation to different stresses by an as yet unknown mechanism.

## Discussion

Over the years, it has been postulated that the correct folding of a protein is dictated mainly by its primary structure. However, it is also known that several human diseases are caused by protein misfolding and aggregation, without any alterations in their primary amino acid sequence (Sauna and Kimchi-Sarfaty, 2011). Hence, other cellular processes that ensure proper protein folding must be considered. Since folding is a co-translational process for many proteins (Thommen et al., 2017), modification of the translation speed of mRNA by synonymous mutations or changes in the abundance or structure of tRNAs could lead to alternative folding, misfolding, and potential aggregation of the encoded protein. Thus codon usage has been proposed as an important determinant of not only protein folding but also efficiency and accuracy of expression and function. Since the influence of codon usage on protein expression, folding and cellular fitness is poorly understood, we studied the effect of synonymous mutations in the gene encoding the glycolytic enzyme Pgk1. To do so, we replaced the WT chromosomal copy of *pgk1* with different mutant versions using a homologous recombination approach, meaning that the mutant proteins would be chronically expressed.

The *pgk1* gene from *S. pombe* is rich in optimal codons (over 90%), as observed for many abundant proteins in other organisms such as *S. cerevisiae*, *E. coli*, and *Candida albicans* (Supplementary Tables 3, 4). It has been reported that codon usage of highly-expressed genes is conserved by natural selection since synonymous mutations could alter processes such as transcription and translation efficiency. For example, Glastad et al. (2015) described that synonymous substitutions may modify DNA methylation patterns of the *pgk1* gene, altering transcription efficiency, and thus gene expression. Also, synonymous substitution could alter translation efficiency by modifying RNA structure, as found in the most common mutation in patients of cystic fibrosis, resulting in a translational pause which impairs the expression and proper folding of the encoded protein (Bartoszewski et al., 2010). Our studies of the effect of synonymous replacements in discrete segments of *pgk1* did not reveal changes in protein levels (Supplementary Figure 1), although we did observe a subtle increase in the mRNA level in one of the mutated strains (Supplementary Figure 2A). This result is opposite to the findings of Hoekema et al. (1987) who found that synonymous mutations in the entire *S. cerevisiae pgk1* gene caused a decrease in mRNA levels attributable to reduced translation speed, which also lowered protein levels. Therefore, we postulate that these effects may vary according to the context and the size of the mutated segments. Evidence supporting this hypothesis is clearly shown as we determined that cell growth is reduced in mutant strains [mut(145–175), mut(177–208) and, to a lesser extent, mut(209–233)] which possess mutations between protein domains (Table 3 and Figure 1). These results agree with previous observation indicating that CUB of regions between protein domains (intrinsically disordered regions) are conserved (low usage codons in general; Zhou et al., 2015). These observations could be explained because such discrete regions might be important in the timing of the folding of domains of the protein, given that the mis-timing of the exit of domains from the ribosome could lead to the abnormal interaction of protein segments. In other studies, differential effects depending on the extent and localization of synonymous mutations have been reported. For example, in experiments carried out in cell free extracts from *Neurospora crassa* (Yu et al., 2015), researchers found that synonymous mutations in a long segment of the luciferase gene (replacing rare codons for their optimal counterparts) increased luciferase activity (as a result of the increase in luciferase expression), although synonymous replacements on short segments within this region showed that not all mutations caused changes in protein expression. Hence, the position of synonymous codons could be determinant for the correct folding of the encoded protein. Moreover, it was found that mutations in different short segments of the luciferase gene produce a less marked effect compared with the mutation of all segments at the same time (Yu et al., 2015). Recently, Walsh et al. (2020) showed that synonymous substitutions in the gene encoding chloramphenicol acetyltransferase alter the co-translational folding of the protein. These changes produced a protein that was more susceptible to degradation, even though it was properly folded and active. Therefore, intermediates in the folding of the protein are probably more prone to degradation.

This evidence suggests that synonymous substitutions could produce subtle changes, depending on the mutated regions, impacting protein biogenesis in different ways (gene expression, mRNA stability, and/or translation rate), and producing different translational effects. It has been suggested that decreasing the translation elongation rate by the substitution of optimal codons with synonymous but rare codons, might give more time to nascent proteins to adopt a stable tertiary structure before the protein is completely synthesized by the ribosome (Hanson and Coller, 2018). This alteration of the translation rate can affect protein folding positively or negatively (Jacobson and Clark, 2016). Our results reveal that subtle changes in both secondary structure as well as activity of Pgk1 were observed in the synonymous mutants, in a region-specific manner (Figure 3). Nevertheless, protein aggregation increased in all mutant strains assayed (Figure 4A). As previously discussed, we consider that synonymous mutations in the regions studied could lead to the generation of different intermediates in the folding process, shaped by different translational kinetics that may alter the exit of the nascent peptide, exposing hydrophobic regions that increase protein aggregation (Horwich, 2002).

Although aggregation levels represent a small fraction (5–10%) of the total Pgk1 protein (Figure 4A), this enzyme is one of the most abundant proteins, with 310,000–560,000 molecules per cell in *S. cerevisiae* (Ghaemmaghami et al., 2003; Kulak et al., 2014). Based on such figures, the absolute number of misfolded molecules in the present study should be approximately 15,000–50,000 molecules per cell. Although protein chaperones are critical to the successful folding of many protein complex (Pechmann and Frydman, 2014; Bagchi et al., 2016; Kramer et al., 2019; Liu et al., 2019), we believe that in the mutant cells, the large amount of misfolded Pgk1 overcomes the function of chaperones. These numerous aggregated molecules may also interact with other proteins and interfere with various processes, for example increasing the malfunction or even triggering aggregation of other proteins interacting with misfolded stretches of Pgk1.

As the expression of mutant Pgk1 is chronic in these cells, this could lead to an adaptation to the stress caused by misfolded proteins, potentially causing the effects observed at the physiological level (Geiler-Samerotte et al., 2011) in response to different stresses, such as heat shock conditions (Figures 4C, 5). Although in the cells carrying mutant Pgk1, the levels of Hsp 16 and 9 chaperone mRNAs were lower than in the control, the response to bortezomib and the increased proteasome activity indicate possible compensatory mechanisms to improve the adaptation of the cells to misfolded proteins (Figures 4D,E). These contradictory observations could be the result of hormetic effects (Zemva et al., 2017), among other factors, involving compensatory events such as potential increases in transcription of those genes encoding proteasome proteases. Similarly, synonymous mutations of codons present in six highly-expressed genes in *E. coli* were shown to alter the overall translation efficiency of the rest of the transcriptome, due to the heightened demands on the translation machinery (Banks et al., 1979; Bowler, 2013; Hoffman et al., 2015). It has also been reported that aged organisms show depressed chaperone levels and activity (Norry and Loeschcke, 2003; Soti and Csermely, 2007) due to damaged proteins, impaired protein synthesis, and other alterations. Thus, *S. pombe* strains harboring the synonymous mutations could be more resistant to stresses due to a premature aging process produced by chronic Pgk1 aggregation.

Pgk1 is not exclusively a glycolytic enzyme; Pgk1 is also found in the nucleus and, during hypoxia, in mitochondria where it participates in DNA replication and regulation of the tricarboxylic acid cycle, respectively (Sun et al., 2015; Li et al., 2016). As several enzymes that participate in the phosphorylation of small metabolites, Pgk1 also function as a protein kinase in a number of fundamental cellular processes. This effect is known as moonlighting (reviewed in Lu and Hunter, 2018) Therefore, other Pgk1 functions are potentially affected by aggregation. Such multiple roles of Pgk1 must be considered in order to understand the effects of synonymous mutations in *pgk1* on cellular response to stresses, as a measurement of cell fitness. It has been reported that in humans, Pgk1 deficiency is an X-linked metabolic disorder produced by a non-synonymous mutation in *pgk1* that leads to altered function or even misfolding (Zoller, 1991). Therefore, the conserved CUB of *pgk1* across the phylogenetic tree could be important in the regulation of *pgk1* expression and function, where any synonymous substitution in the coding sequence could exert a pleiotropic effect, as observed in this work. Because the many functions of Pgk1, it would be very important to analyze the effect of synonymous mutations in *pgk1* from different organisms as other yeast as well as more complex organisms such as mammals and even humans. The data obtained in this work led us to predict that synonymous mutations might exert changes in the physiology of these organisms at different levels.

## Conclusion

The replacement of preferred codons by rare codons in discrete segments along the entire sequence of *pgk1* from *Schizosaccharomyces pombe* generated subtle differences in the function of the expressed protein, particularly in the activity of the enzyme. However, irrespective of the location, every mutated segment induced aggregation of the protein. Therefore, the chronic expression of altered Pgk1 may make the cells permanently exposed to stress and thus more resistant to additional stresses.

## Materials and methods

### Calculation of codon and tRNA adaptation indexes

tRNA (tAI) and codon (CAI) adaptation indexes were calculated as described previously (Sharp and Li, 1987; Mehdi et al., 2014), using the data available in http://gtrnadb2009.ucsc.edu/ and http://www.kazusa.or.jp/codon/, respectively.

### Yeast strains and media

The *Schizosaccharomyces pombe* 972 h- (ATCC 24843) strain was used for homologous recombination experiments. Primers and plasmids used in this study are listed in Supplementary Table 1. *S. pombe* was grown on YES medium (5 g/l yeast extract, 30 g/l glucose), YPD (10 g/l yeast extract, 20 g/l peptone, 20 g/l glucose), and EMM2 minimal medium (US Biological) supplemented with 20 g/l glucose.

### Plasmid construction

The synonymous substitutions were performed based on *S. pombe* codon usage, replacing optimal codons with their non-optimal counterpart (according to Forsburg, 1994). The endogenous *pgk1* gene was replaced by homologous recombination with the wild-type or mutated *pgk1*, both with a 7xHis-tag at the 3′ end. For homologous recombination, the flanking regions and *pgk1* coding sequence were cloned in the pFA6a-KanMX6 vector (Addgene), as described below. Flanking regions were amplified from *S. pombe* genomic DNA (gDNA), using Herculase II Fusion DNA polymerase (Agilent Genomics) according to the manufacturer’s instructions. For the 5′ flanking region, amplification was performed in two rounds of PCR (Zoller, 1991) to eliminate an internal restriction site (NdeI), using 5′F and 5′R-intern and 5′F-intern and 5′R primers (Supplementary Table 1). For the 3′ flanking region, 3′F and 3′R primers were used (Supplementary Table 1). PCR products were cloned in pGEMT-easy (Promega, United States) and the recombinant plasmids were digested with PfoI and NdeI (for the 5′ flanking region) and SacI and EcoRI (for the 3′ flanking region). The digestion products were ligated into pFA6a-KanMX6 (first the 5′ flanking region, followed by the 3′ flanking region), that had been previously digested with the corresponding restriction enzymes. The final vector was named pFA6a-KanMX6-5′-3′ and confirmed by DNA sequencing. The WT *pgk1* coding sequence was amplified from gDNA using the following primers: PGK1-F and PGK1-R (with a 7xHis tag sequence at the 3′ end) (Supplementary Table 1). The product was cloned into pGEMT-easy, and the construction was used as template for the amplification of the WT and mutant sequences. All *pgk1* mutants were constructed as follows: Firstly, the amplification of the mutant *pgk1* gene sequence was carried out in two separate PCR reactions using (i) PGK1-F primer and the corresponding reverse internal primer containing the mutated codons (for example, for the mutation of codons 112–142, the corresponding primer was 112–142-R) (Supplementary Table 2) to amplify one segment (in this case, 112–142-i) and (ii) PGK1-R and the corresponding forward primer containing the mutated codons (for the same example, 112–142-F) to amplify the second fragment (in this case, 112–142-ii). PCR products were purified and then used as template for two separate amplifications, where 112–142-i was amplified with the PGK1-F primer, and 112–142-ii was amplified with the PGK1-R primer. Secondly, both products were mixed and incubated in a thermocycler in the presence of 0.2 mM dNTPs, 1X Herculase II Fusion DNA polymerase buffer, and Herculase II Fusion DNA polymerase (30 cycles of: 30 s at 95°C, 30 s at 30°C; 1 min at 72°C). Thirdly, the final product was purified and digested with NdeI and BamHI, and ligated into pFA6a-KanMX6-5′-3′. Finally, *E. coli* JM109 strain was transformed with the corresponding construction by chemical transformation (Chung et al., 1989) and 3–5 clones were analyzed by DNA sequencing to corroborate the correct incorporation of mutations.

### Homologous recombination

Purified plasmids containing the flanking regions plus the His-tagged WT and mutant coding sequences of *pgk1* were used for amplification of the sequence employed for homologous recombination, using 5′F and 3′R primers (Supplementary Table 1). The corresponding PCR product (100 ng) was electroporated into 100 μl electrocompetent *S. pombe* prepared as described previously (Forsburg and Rhind, 2006). Yeasts were recovered in 1 M sorbitol at 30°C for 3 h, and then cells were pelleted, resuspended in 200 μl YE medium, and plated on YPD agar in the presence of 200 μg/ml G418 antibiotic (Sigma-Aldrich). Cells were grown for 4–5 days at 30°C and single colonies were picked and grown on YPD medium supplemented with 200 μg/ml G418 antibiotic. To corroborate the incorporation of synonymous mutations, the His-tagged *pgk1* coding sequence (WT and mutants) were sequenced after amplification of the gDNA of 5–15 clones for each mutated region, using PGK1-F and PGK1-R primers. Additionally, to check that the recombination occurred in the endogenous *pgk1* gene, we designed primers that amplify the locus segment outside the recombined segment (Supplementary Table 1), Forward_Out_PGKF and Reverse_Out_PGKR, combined with internal primers within the segment.

### Growth assays

Yeasts were grown overnight in EMM2 medium at 30°C. Then, cells were pelleted (2,250 × *g* for 5 min) and resuspended in EMM2 at 0.05 OD600 (measured in an Infinite 200 PRO microplate plate reader, TECAN). Yeast cultures (200 μl) were inoculated in a 96-well plate (in triplicate), and cells were incubated for 24 h in constant agitation at 30°C in the plate reader, recording OD600 each hour. For thermal stress, cells were incubated at 42°C for 2 h, and then at 37°C for 22 h. For growth in the presence of the proteasome inhibitor Bortezomib (Santa Cruz Biotechnology), cells were prepared as previously described at a final OD600 of 0.05. Bortezomib was then added at a final concentration of 100 μM, or an equal quantity of DMSO (solvent) and cells were inoculated in 96-well plates in triplicate for each condition. Cells were grown for 24 h, and the OD600 was recorded. For the specific growth rate (μ index), the OD600 at the exponential phase of each strain was determined, and the values inserted into the following equation:


$$k=\frac{\ln\left(\frac{N2}{N1}\right)}{t2-t1}$$

where *k* is the specific growth rate, N1 and N2 are the initial and terminal OD600 recordings, respectively, and t1 and t2 correspond to initial and terminal times, respectively (in hours). Thus, *k* is expressed in h^−1^. The generation (or doubling) time was calculated according to the following equation:


$$Generationtime:\frac{\ln\left(2\right)}{k}$$

where generation time is expressed in hours.

### Messenger RNA isolation and quantification

For *pgk1* mRNA quantification, WT and mutant strains were grown to the late exponential phase (OD600, 1.0) in 20 ml EMM2 under standard conditions. For chaperone mRNA quantification, cells were grown in 20 ml EMM2 for 24 h under standard conditions or under heat stress, as described above. Yeasts were then pelleted (2,250 × *g* for 5 min) and washed once with sterilized water. The cell wall was disrupted with 0.12 μg zymolyase 20 T (US Biological) in 1 M sorbitol for 30 min at 37°C. Cells were pelleted and resuspended in 200 μl TRIzol (Thermo Scientific) and vortexed three times for 1 min each (intercalated with 1 min on ice). Then, 40 μl chloroform was added to the mix and immediately centrifuged at 12,000 × *g* for 20 min at 4°C. The supernatant was precipitated with 0.7 volumes of isopropanol at –80°C overnight. RNA was pelleted at 15,000 × *g* for 30 min and washed once with 80% ethanol. RNA was resuspended in 30 μl sterilized miliQ water and quantified in a nanodrop (BioTek), then visualized in an 1% agarose gel to check integrity. RNA (1 μg) was treated with DNAseI (Roche), according to the instructions provided in the manual, and then 500 ng RNA were used for cDNA synthesis followed by Real-Time PCR with the Brilliant II QRT-PCR, AffinityScript Two-Step Master Mix (Agilent), according to manufacturer instructions. The primers used for Real-Time PCR are listed in Supplementary Table 1 (PGK1RT-F and PGK1RT-R for *pgk1* amplification). Quantifications were performed using *actin* as a control (ActRT-F and ActRT-R for actin amplification).

### Isolation and quantification of total and aggregated protein

WT and mutant strains were grown to the exponential phase (OD600, 1.0) in 20 ml EMM2. Yeasts were harvested by centrifugation (2,250× *g* for 5 min), and the pellet was used to analyze protein aggregation as described previously (Rand and Grant, 2006). Briefly, spheroplasts were pelleted and resuspended in lysis buffer [50 mM potassium phosphate buffer, pH 7, 1 mM EDTA, 5% glycerol, 1 mM phenylmethylsulfonyl fluoride and Complete Mini protease inhibitor cocktail (Roche)]. Cell disruption was carried out by three vortex cycles (1 min of vortex and 1 min on ice) with 220 mg of acid-washed glass beads (Sigma-Aldrich G8772). Membrane proteins were removed by washing twice with 320 μl lysis buffer and 80 μl 10% NP-40 (Sigma-Aldrich), and the final aggregated protein extract was resuspended in 20 μl 1X loading buffer. Total and aggregated protein extracts were analyzed by Western blotting using an anti His-tag antibody (His Tag Antibody MAB050R-100, R&D Systems). Western blots against tubulin were performed as internal controls using T5168 monoclonal anti-a-tubulin clone B-5-1-2 (Sigma-Aldrich). Bands corresponding to WT, mutants, and tubulin (for total protein), or WT and mutants (for aggregated proteins) were quantified using ImageJ software.

### Protein purification

Yeasts (WT and mutants) were grown at 30°C until the stationary phase, and then soluble proteins were isolated as described previously (Rand and Grant, 2006), scaled to a 160 ml culture (EMM2). Soluble protein was incubated for 2 h with 250 μl Ni-PentaTM Agarose 6 fast flow resin (Marvelgent Biosciences Inc), previously equilibrated with lysis buffer at 4°C. The resin was then washed with 20 volumes of washing buffer (20 mM HEPES pH 7.0, 300 mM NaCl, 1 mM β-mercaptoethanol) supplemented with 2 mM imidazole. His-tagged proteins were eluted with 10 volumes of elution buffer (washing buffer supplemented with 25 mM imidazole), and collected in 13 fractions (100 μl each). Fractions were analyzed by SDS-PAGE, and fractions 2–8 were selected to be dialyzed with the corresponding buffer for all yeasts assayed.

### Circular dichroism

Purified proteins (WT and mutants) were dialyzed against 20 mM phosphate buffer, pH 7.0, at 4°C overnight. Purified proteins (300–500 mg/ml) were used for CD analyses in a Jasco J-1500 spectrometer. CD spectra were recorded between 200 and 250 nm, at a data pitch of 1.0 nm and a scanning speed of 50 nm/min. Three accumulations were obtained for each measurement, and the data were processed with the following equation:


$$\left[\theta \right]=\frac{mdeg*molecularweight}{10*\left[\frac{mg}{ml}\right]*l*numberofaminoacid}$$

### Enzyme activity

Purified WT and mutant proteins were dialyzed against activity buffer (25 mM Tris–HCl, pH 7.4, 20% glycerol, 0.5 mM MgCl_2_, 1 mM phenylmethylsulfonyl fluoride) at 4°C overnight. The activity assay was carried out with 5 nM of each protein, in the presence of 7 mM 3-PGA, 3 mM ATP, 1 mM MgCl_2_, 50 mM Tris–HCl pH 7.4, and 0.5 mM NADH, in a final volume of 200 μl. The reaction mixture was placed in a 96-well plate and incubated at 25°C, measuring the absorbance at 340 nm in an Infinite 200 PRO microplate plate reader every 15 s.

### Proteasome activity

Proteasome activity was measured with the Proteasome Activity Assay Kit (Fluorometric) (Abcam, ab0107921). Total protein extracts were obtained as described previously (Rand and Grant, 2006) from 5 ml culture of each strain (OD600 1.0). Then, 1 mg of each protein extract was mixed with Assay Buffer in a final volume of 100 μl, in independent wells of a 96-well dark plate (for fluorometric assays), in triplicate. Proteasome substrate (1 μl, 5 mM) was added to each well, and then proteasome activity was determined as the change in fluorescence, measuring excitation/emission at 350/440 nm in the Infinite 200 PRO microplate plate reader at 37°C for 25 min. A calibration curve was also generated, as indicated by the manufacturer, in order to determine the product concentration in the assays. One unit of proteasome activity corresponds to the amount of proteasome which generates 1 nmol of product per minute at 37°C.

## Data availability statement

The original contributions presented in the study are included in the article/Supplementary material, further inquiries can be directed to the corresponding author.

## Author contributions

SM-R designed and performed most of the experiments and participated in writing the manuscript. LA carried out some experiments and discussed results. AK participated in the design and discussion of results. RF participated in the discussion and writing. GL performed some experiments and discussed results. OO provided the funding, designed the experimental strategy, discussed results, and wrote the manuscript. All authors contributed to the article and approved the submitted version.

## Funding

This work was funded by FONDECYT, Chile Postdoctoral 2014 Grant 3150366 (SM-R) and FONDECYT Regular Grants 1110834 and 1190552 (OO), 1211386 (GL), 1191074 (AK) and Universidad de Chile and Universidad de Santiago de Chile. LA was the recipient of a fellowship for graduate studies from CONICYT, Chile.

## Conflict of interest

The authors declare that the research was conducted in the absence of any commercial or financial relationships that could be construed as a potential conflict of interest.

## Publisher’s note

All claims expressed in this article are solely those of the authors and do not necessarily represent those of their affiliated organizations, or those of the publisher, the editors and the reviewers. Any product that may be evaluated in this article, or claim that may be made by its manufacturer, is not guaranteed or endorsed by the publisher.
