# Supplementary material for: Synonymous mutations in the phosphoglycerate kinase 1 gene induce an altered response to protein misfolding in Schizosaccharomyces pombe
Source: Front Microbiol. 2023 Jan 11;13:1074741. doi: 10.3389/fmicb.2022.1074741 (PMC9875302; doi:10.3389/fmicb.2022.1074741)
Supplement: Supplementary file 1 [file Data_Sheet_1.PDF]

Supplementary Figures

```
S.cerevisiae    -SLSSKLSVQDLDLKDKRVFIRVDFNVPLDGKKITSNQRIVAALPTIKYVLEHHPRYVVL
S.pombe         MSLSTKLAITDVDLKGKNVLIRVDFNVPLDGDRITNNARIVGALPTIKYALEQQPKAVIL
H.sapiens       MSLSNKLTLDKLDVKGKRVVMRVDFNVPMKNNQITNNQRIKAAVPSIKFCLDNGAKSVVL
                 ***.**:: .:*:*.*.*.:*******:...:**.* ** .*:*:**: *:: .: *:*
S.cerevisiae    ASHLGQPNGERN-EKYSLAPVAKELQSLLGKDVTFLNDCVGPEVEAAVKASAPGSVILLE
S.pombe         MSHLGRPNGARV-AKYSLKPVAAELSKLLGKPVKFLDDCVGPEVEKACKEAKGGEVILLE
H.sapiens       MSHLGRPDGVPMPDKYSLEPVAVELKSLLGKDVLFLKDCVGPEVEKACANPAAGSVILLE
                 ****:*:*     **** *** **..**** * **.******** *   .  *.*****
S.cerevisiae    NLRYHIEEEGSRK-VDGQKVKASKEDVQKFRHELSSLADVYINDAFGTAHRAHSSMVGFD
S.pombe         NLRFHIEEEGSAK-VDGKKVKADASAVEAFRKSLTSLGDIFVNDAFGTAHRAHSSMVGVD
H.sapiens       NLRFHVEEEGKAKDASGNKVKAEPAKIEAFRASLSKLGDVYVNDAFGTAHRAHSSMVGVN
                ***:*:****. * ..*:****.   :: ** .*:.*.*:::****************.:
S.cerevisiae    LPQRAAGFLLEKELKYFGKALENPTRPFLAILGGAKVADKIQLIDNLLDKVDSIIIGGGM
S.pombe         LP-RVSGFLMKKELDYFSKALENPARPFLAILGGAKVADKIQLIDNLLDKVNRLIICGGM
H.sapiens       LPQKAGGFLMKKELNYFAKALESPERPFLAILGGAKVADKIQLINNMLDKVNEMIIGGGM
                ** :..***::***.**.****.* *******************:*:****: :** ***
S.cerevisiae    AFTFKKVLENTEIGDSIFDKAGAEIVPKLMEKAKAKGVEVVLPVDFIIADAFSADANTKT
S.pombe         AFTFLKVLNGMKIGDSLFDEAGSKNVESMMAKAKKNNVEVFLPVDFVTADKFDKDAKVGS
H.sapiens       AFTFLKVLNNMEIGTSLFDEEGAKIVKDLMSKAEKNGVKITLPVDFVTADKFDENAKTGQ
                **** ***:. :** *:**: *:: * .:* **: :.*:: *****: ** *. :*:.
S.cerevisiae    VTDKEGIPAGWQGLDNGPESRKLFAATVAKAKTIVWNGPPGVFEFEKFAAGTKALLDEVV
S.pombe         ATAEEGIPDGWMGLDCGPKSSAKFAEVITTSKTIVWNGPAGVFEFDNFAKGTKSMLDACV
H.sapiens       ATVASGIPAGWMGLDCGPESSKKYAEAVTRAKQIVWNGPVGVFEWEAFARGTKALMDEVV
                .*  .*** ** *** **:*   :* .:: :* ****** ****:: ** ***:::*  *
S.cerevisiae    KSSAAGNTVIIGGGDTATVAKKYGVTDKISHVSTGGGASLELLEGKELPGVAFLSEKK
S.pombe         KTCEAGNVVIVGGGDTATVAKKYGKEDALSHVSTGGGASLELLEGKALPGVVALSSK-
H.sapiens       KATSRGCITIIGGGDTATCCAKWNTEDKVSHVSTGGGASLELLEGKVLPGVDALSNI-
                *:   *  .*:******* . *:.  * :***************** ****  **.
```

Supplementary Figure 1. Alignment of the Pgk1 protein sequences from different sources. Symbols represent identical (*), chemically similar (:) and similar in size (.) amino acids, respectively

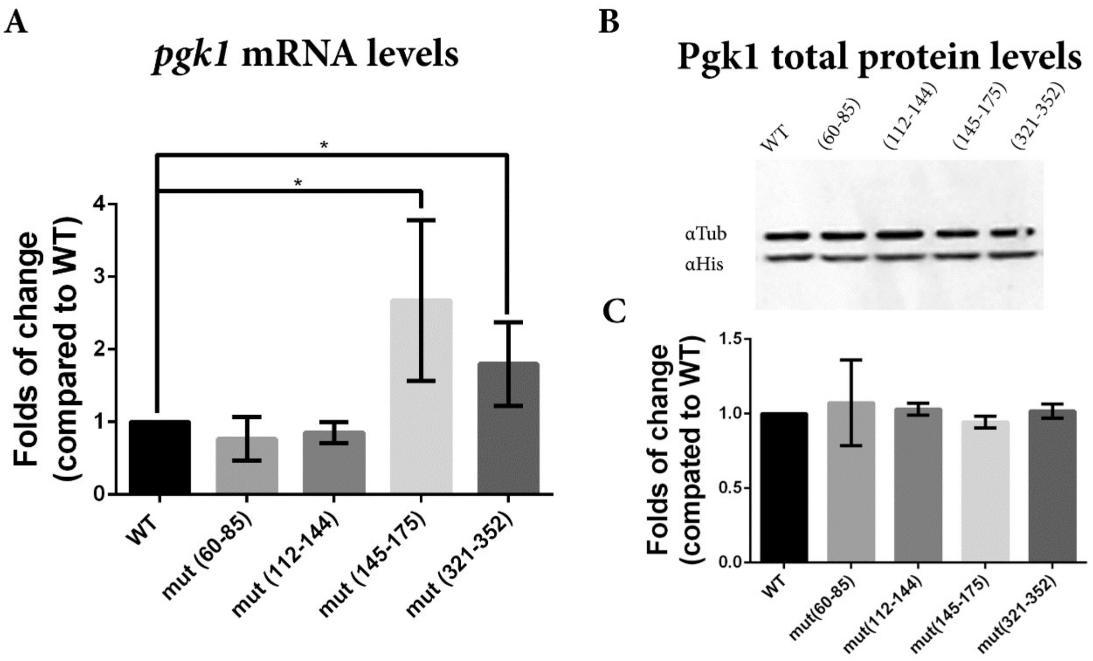

Supplementary Figure 2. Synonymous mutation in selected codons did not alter the expression of Pgk1. (A) Quantification of *pgk1* mRNA levels in WT and mutant strains cultured in EMM2 at stationary phase in standard conditions. Normalization was performed using *actin* mRNA levels, and then compared to WT. * $p<0.05$. **(B)** Western blot of Pgk1 (αHis) and tubulin (αTub) from total proteins extracted from yeasts cultured in EMM2 medium and harvested during the late logarithmic phase in standard conditions. **(C)** Quantification of Pgk1 levels, normalized to tubulin and presented as percentage compared to WT. Data correspond to three independent experiments analyzed by one-way ANOVA.

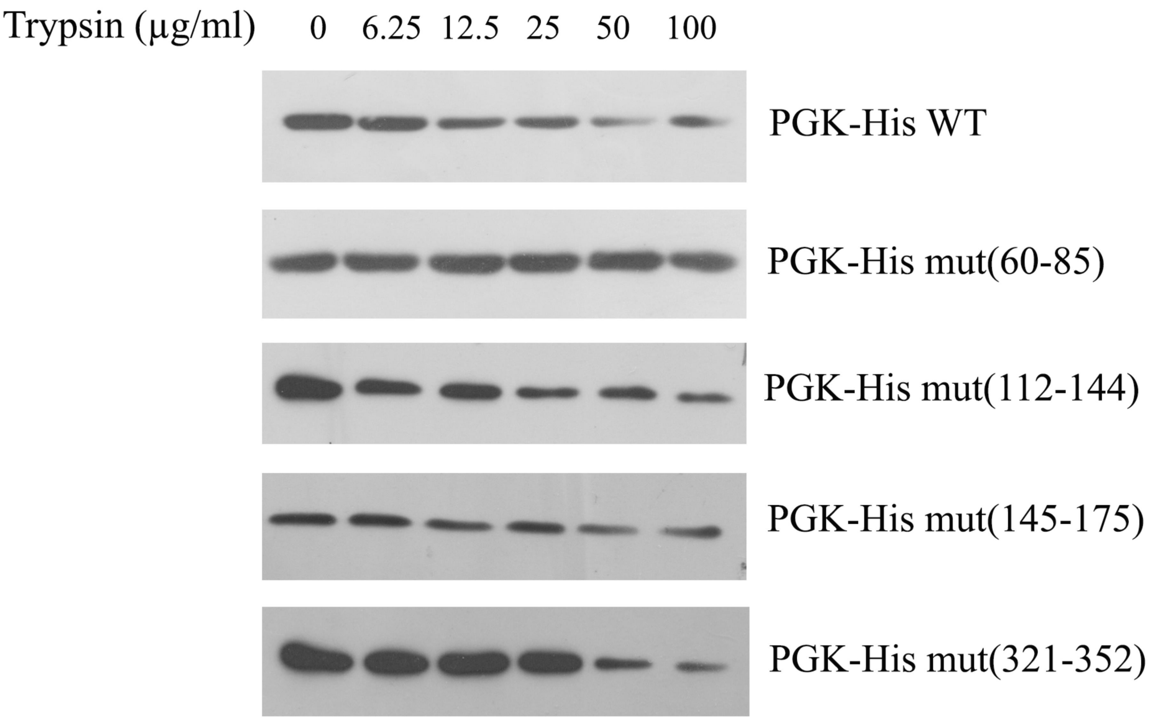

Supplementary Figure 3. Extracts from the strains carrying the corresponding mutations were exposed to trypsin at the indicated concentrations for 6 min at 37^0C. Then the reactions were stopped by the addition of soybean inhibitor and heated at 95^0C for 4 min. Samples were subjected to SDS-PAGE and analyzed by Western blot using anti-His antibody.

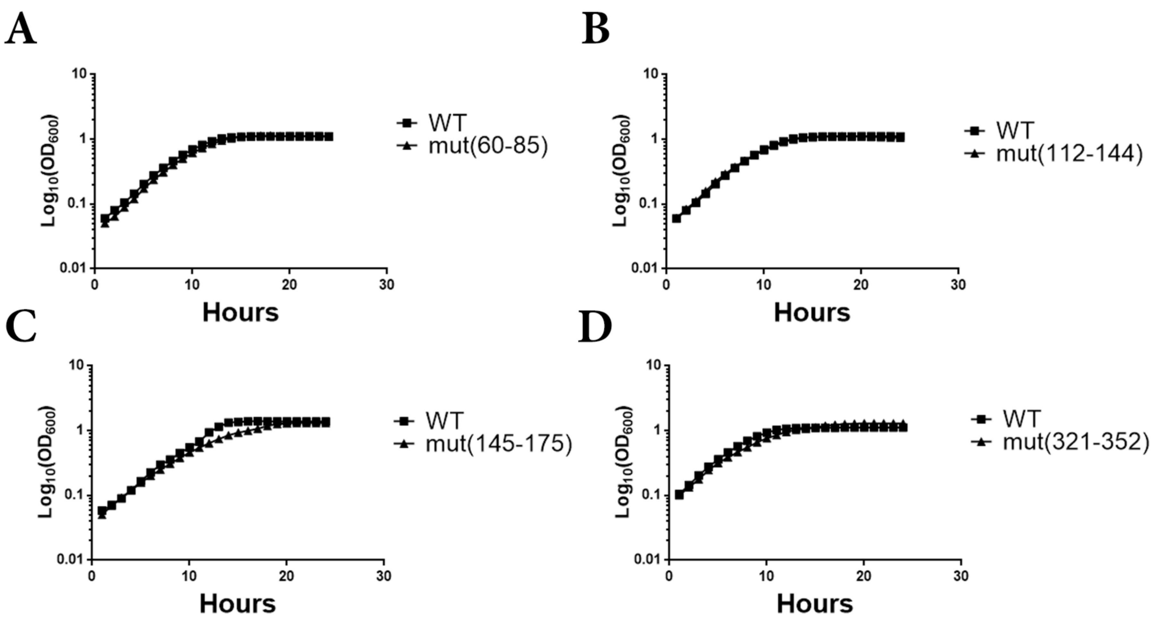

Supplementary Figure 4.

Yeast growth in minimal medium is altered in certain strains harboring synonymous mutations. Yeasts were grown in minimal medium (EMM2) under standard conditions for 24 hours at 30°C, and OD_{600} recorded each hour. Readings of (A) mut(60-85), (B) mut(112-142), (C) mut(145-175) and (D) mut(321-352) were plotted and compared to the control (WT). These results are representative of three independent experiments.

This figure is the new version of Figure 2 where the scale of Y axis was replaced by a logaritmic scale.

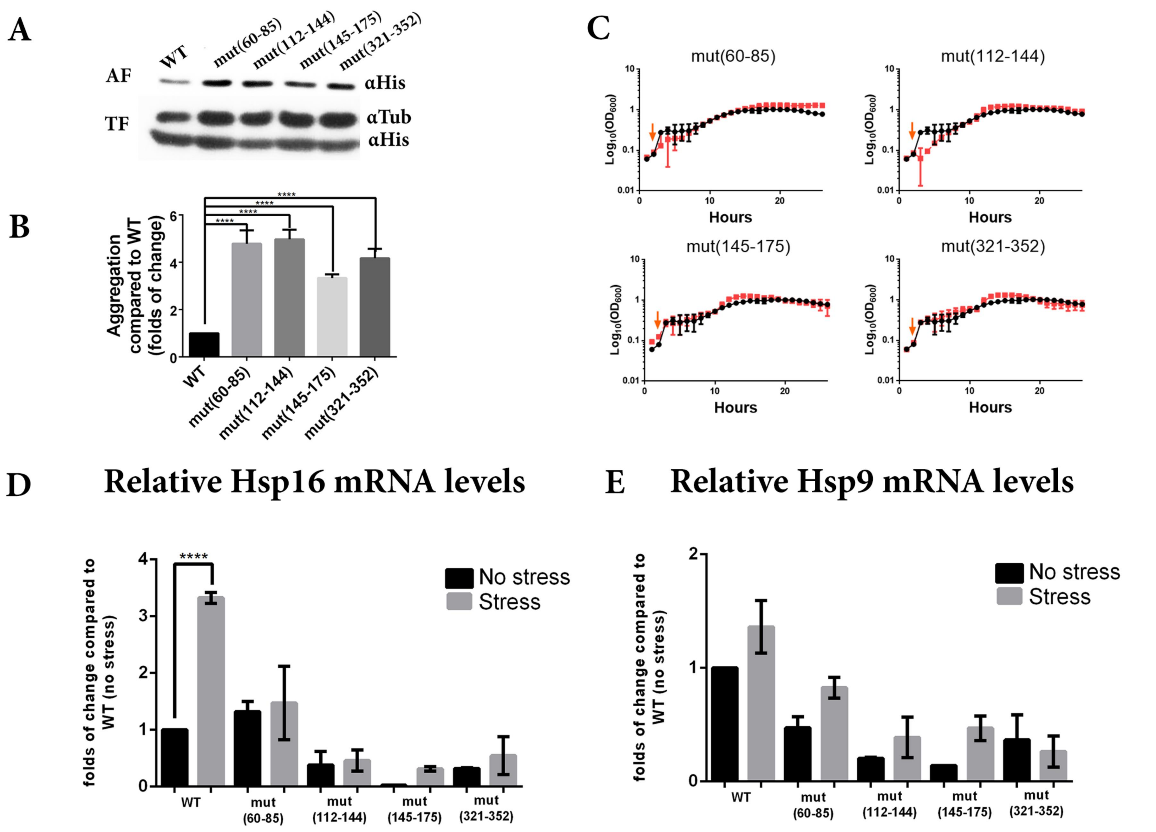

Supplementary Figure 5. Synonymous mutations in *pgk1* alter protein aggregation, response to heat stress and chaperone expression. (A) Western blot of WT and mutants (αHis) from aggregated (AF) and total (TF) protein fractions isolated from yeasts cultured in EMM2 during the late logarithmic phase in standard conditions. (B) Quantification of the aggregated fraction (aggregated Pgk1 normalized to total Pgk1) and expressed as fold-change compared to WT. Data was analyzed by one-way ANOVA (**** p<0.0001). (C) Response to heat stress. Yeasts were grown in EMM2 at 42°C for two hours, then shifted to 37°C (chronic heat stress, indicated with orange arrow) for 24 hours. Black circles and red squares represent WT and mutant strains respectively. (D and E) Quantification by real-time PCR of the levels of chaperones Hsp16 (D) and Hsp19 (E) mRNAs from WT and mutants, grown under standard conditions (30°C for 24 hours, no stress, black bars) or heat stress condition (2 hours at 42°C and 24 hours at 37°C, stress, gray bars), The results are representative of three independent experiments, and data was analyzed by two-way ANOVA (p<0.005).

This figure is the new version of Figure 4 where the scale of Y axis in panel C was replaced by a logaritmic scale.

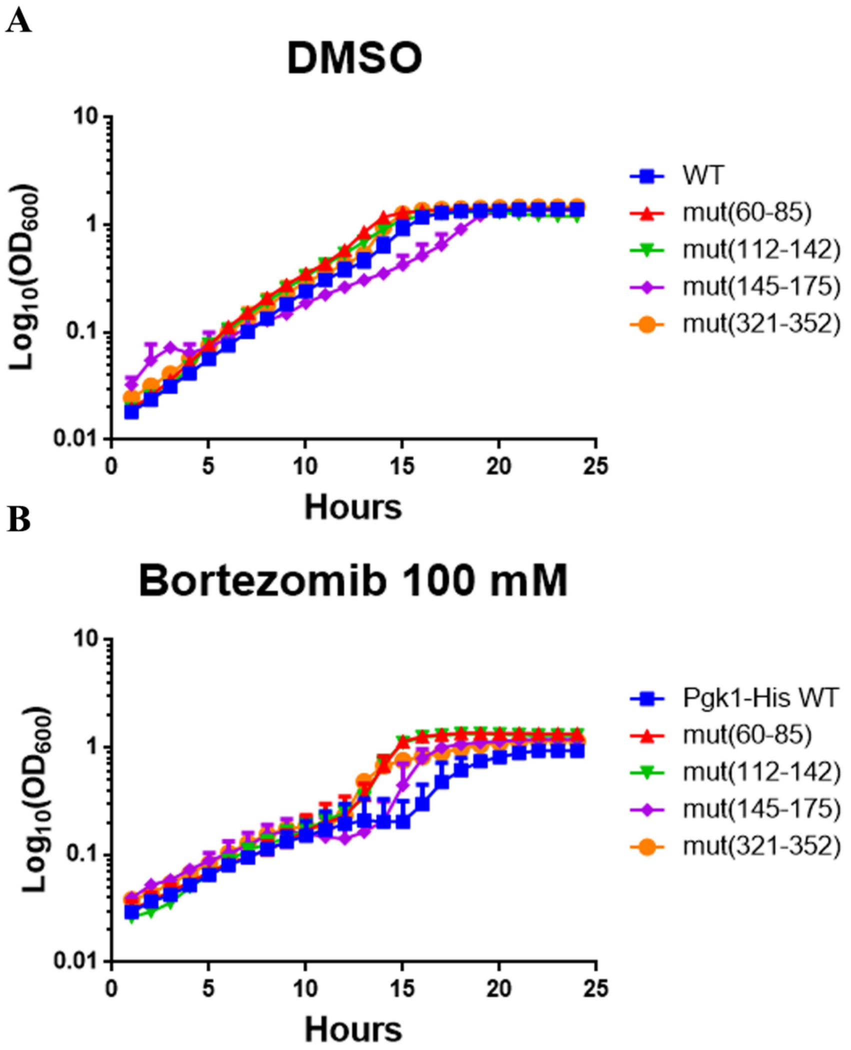

Supplementary Figure 6. Response to the proteasome inhibitor bortezomib is improved by synonymous mutations in *pgk1*. Yeasts were incubated with vehicle (DMSO) (A) or 100 µM bortezomib (B) in EMM2 for 24 hours in standard conditions. Blue squares, red triangles, green inverted triangles, purple diamonds and orange circles represent WT, mut(60-85), mut(112-144), mut(145-175) and mut(321-352) respectively. Growth was monitored by recording OD_{600} at each hour.

This figure is the new version of Figure 5 where the scale of Y axes were replaced by logaritmic scales.

Supplementary Tables

Supplementary Table 1. List of primers used for amplification of 3’ and 5’ flanking regions and *pgk1* coding sequence. Digestion sites were added at the 5’ end of the corresponding primer.

Name	Sequence	Digestion site
3'F	GAGCTCACTTCCTTCTCAATGCC	*Sac*I
3'R	GAATTCATAGGAAAGGAATGAGAATAATATCC	*Eco*RI
5'F	TCCCGGAAACGCTACTTGATCGG	*Pfo*I
5'R intern	CCGTGGATCATTTGTATGTTTC	None
5'F intern	GAAACATACAAATGATCCACGG	None
5'R	CATATGTATGTGATTGATTGATTC	*Nde*I
PGK1-F	GCACTACATATGTCTTTGTCTACTAAGCTCG	*Nde*I
PGK1-R	GCACTGGATCCTTAATGATGGTGATGATGG	*Bam*HI
Forward_Out_PGKF	GTTACGAAGCGATAGTAGATAGC	None
Reverse_Out_PGKR	GGATAGAGCAACGCGTAACAG	None
ActRT-F	CGGTCGTGACTTGACTGACT	None
ActRT-R	TCAAGGGAGGAAGATTGAGC	None
PGK1RT-F	TATCACCACCTCCAAGACCA	None
PGK1RT-R	TGGCAACAGTAGCAGTGTCA	None

Supplementary Table 2. List of primers used for amplification of mutant sequences. The name of the corresponding primers is indicated in the left-hand column (F: forward primer; R: reverse primer). The primers were designed according to IUPAC nomenclature. The names of the strains (as shown in Table 1) produced by the synonymous mutations made with the corresponding pair of primers are listed in the right-hand column.

Name	Sequence	Name of mutated strain
60-85-F	CGTGTTGCCAAATATTCDTTAAAACCVGTVGCVGCVGARCTCAGC	mut(60-85)
60-85-R	GGCAACACGBGCBCCRTTHGGYCKHCCTAAATGYGACATCAA	mut(60-85)
112-144-F	GARGAGGAGGGTTCNGCDAAAGTVGAYGGVAARAARGTVAARGCVGAYGCVTCRGCD	mut(112-144)
112-144-R	GAACCCTCCTCYTCWATATGAAANCGYARATTYTCYARBAGWATHACYTCBCCBCC	mut(112-144)
145-175-F	ATCTTTGTCAAYGAYGCVTTYGGVACDGCVCAYCGVGCVCAYTCDTCVATGGTC	mut(145-175)
145-175-R	GACAAAGATRTCBCCHAGBGABGTNAGBGATTTBCGRAABGCYTCGAC	mut(145-175)
321-352-F	GGTCCCGCTGGVGTDTTYGARTTYGAYAAYTTYGCDAAAGGVACDAAATCTATG	mut(321-352)
321-352-R	AGCGGGACCRTTCCAHACDATGGTTTTHGAHGTHGTWATBACYTCHGCAAA	mut(321-352)
34-59-F	CCCACCATCAAATATGCRYTVGAACAACAGCCDAAAGCRGTCATC	mut(34-59)
34-59-R	GATGGTGGGHARBGCBCCBACWATBCKYGCGTTATTYGTGAT	mut(34-59)
209-223-F	TGCTGACAAAATWCARCTVATMGACAAC	mut(209-223)
209-223-R	TTGTCAGCAACCTTHGCBCCBCCCAA	mut(209-223)
257-288-F	AAGAACAACGTDGARGTDTTYCTDCCDGTVGAYTTYGTDACVGCDGACAAG	mut(257-288)
257-288-R	GTTGTTCTTTTTHGCTTTHGCCATCATHGAYTCHACRTTTTTHGABCCBGCCTC	mut(257-288)
289-320-F	GGTATCCCCGAYGGNTGGATGGGVTTAGAYTGYGGNCCDAAATCDTCVGCTAAG	mut(289-320)
289-320-R	GGGGATACCCTCYTCHGCHGTHGCBGAHCCBACTTTHGCRTCTTTRTCGAA	mut(289-320)

Supplementary Table 3. Codon Adaptation Index of *pgk1*, showing highly-expressed and less-expressed genes of several species. The Gene ID corresponds to the number of the gene assigned in the NCBI database.

Species	CAI *pgk*1	CAI (highly-expressed gene, Gene ID)	CAI (less-expressed gene, Gene ID)
Escherichia coli	0.72	0.70 (rplB, 947820)	0.25 (dnaG, 947570)
Vibrio cholera	0.73	0.75 (rplB, 2615610)	0.35 (dnaG, 2614953)
Saccharomyces cerevisiae	0.76	0.73 (RPL2A, 850590)	0.057 (CDC13, 851306)
Schizosaccharomyces pombe	0.86	0.79 (rpl802, 2540366)	0.38 (sfc2, 2542887)
Candida albicans	0.49	0.46 (RPL2, 30515049)	0.36 (CDC13, 3639293)
Caenorhabditis elegans	0.43	0.4 (rpl-2, 180343)	0.33 (cyclin A, 186646)

Supplementary Table 4. tRNA Adaptation Index of *pgk1*, showing highly-expressed and less-expressed genes of several species. The Gene ID corresponds to the number of the gene assigned in the NCBI database.

Species	tAI *pgk*	tAI (highly-expressed gene, Gene ID)	tAI (less-expressed gene, Gene ID)
Escherichia coli	0.3025	0.2902 (rplB, 947820)	0.2505 (dnaG, 947570)
Vibrio cholera	0.2992	0.3048 (rplB, 2615610)	0.1960 (dnaG, 2614953)
Saccharomyces cerevisiae	0.4356	0.3612 (RPL2A, 850590)	0.2387 (CDC13, 851306)
Schizosaccharomyces pombe	0.3980	0.3539 (rpl802, 2540366)	0.1625 (sfc2, 2542887)
Candida albicans	0.5789	0.5410 (RPL2, 30515049)	0.3202 (CDC13, 3639293)
Caenorhabditis elegans	0.4840	0.5529 (rpl-2, 180343)	0.3938 (cyclin A)
